# Laxity measurement of internal knee rotation after primary anterior cruciate ligament rupture versus rerupture

**DOI:** 10.1007/s00402-021-04269-1

**Published:** 2021-12-06

**Authors:** Hermann O. Mayr, Georg Hellbruegge, Florian Haasters, Bastian Ipach, Hagen Schmal, Wolf C. Prall

**Affiliations:** 1 Department of Orthopedic and Trauma Surgery, University Hospital, Albert Ludwig University of Freiburg, Hugstetter Str. 55, 79106 Freiburg, Germany; 2 Schoen Clinic Munich Harlaching, Academic Teaching Hospital Munich University, Munich, Germany; 3 Department of General, Trauma and Reconstructive Surgery, University Hospital, Ludwig-Maximilians-University Munich, Munich, Germany

**Keywords:** Rotational laxity, Instrumented measurement, ACL, Anterior cruciate ligament, Rerupture, Anterolateral ligament, ALL

## Abstract

**Purpose:**

The aim of the current study was to objectify the rotational laxity after primary anterior cruciate ligament (ACL) rupture and rerupture after ACL reconstruction by instrumented measurement. It was hypothesized that knees with recurrent instability feature a higher internal rotation laxity as compared to knees with a primary rupture of the native ACL.

**Study design:**

Cross-sectional study, Level of evidence III.

**Methods:**

In a clinical cross-sectional study successive patients with primary ACL rupture and rerupture after ACL reconstruction were evaluated clinically and by instrumented measurement of the rotational and antero-posterior laxity with a validated instrument and the KT1000^®^, respectively. Clinical examination comprised IKDC 2000 forms, Lysholm Score, and Tegner Activity Scale. Power calculation and statistical analysis were performed (*p* value < 0.05).

**Results:**

24 patients with primary ACL rupture and 23 patients with ACL rerupture were included. There was no significant side-to-side difference in anterior translation. A side-to side difference of internal rotational laxity ≥ 10° was found significantly more frequent in reruptures (53.6%) compared to primary ruptures (19.4%; *p* < 0.001). A highly significant relationship between the extent of the pivot-shift phenomenon and side-to-side difference of internal rotation laxity could be demonstrated (*p* < 0.001). IKDC 2000 subjective revealed significantly better scores in patients with primary ACL tear compared to patients with ACL rerupture (56.4 ± 7.8 vs. 50.8 ± 6.2; *p* = 0.01). Patients with primary ACL tears scored significantly better on the Tegner Activity Scale (*p* = 0.02). No significant differences were seen in the Lysholm Score (*p* = 0.78).

**Conclusion:**

Patients with ACL rerupture feature significantly higher internal rotation laxity of the knee compared to primary ACL rupture. The extend of rotational laxity can be quantified by instrumented measurements. This can be valuable data for the indication of an anterolateral ligament reconstruction in ACL revision surgery.

## Introduction

Injuries with tears of the anterior cruciate ligament (ACL) result in anterior to posterior (a.p.) translational and internal rotational instability of the knee joint. The extend of internal rotational laxity depends on individual anatomical characteristics (general joint laxity [37], tibial slope, femoral condyle shape [37], and mechanical alignment) as well as the traumatic lesions of anterolateral and intraarticular structures [menisci, iliotibial band, Kaplan fibers, capsule, and the anterolateral ligament (ALL)]. The ALL is a crucial peripheral rotational stabilizer of the knee joint [7, 8, 33], and has increasingly been investigated on in clinical context over the last years [10, 34, 41, 48, 49]. In cases of pronounced anterolateral rotational instability of the knee joint, a growing number of publications postulate a simultaneous reconstruction of the ALL and the ACL [15, 16, 22, 32, 36, 38]. Various operative techniques have been proposed [13, 15, 19, 35, 43]. Knees with persistent internal rotational instability may be more prone to suffer a reruptur secondary to the ACL reconstruction as ongoing pivot-shift phenomenon and rotational laxity has been shown to correlate with inferior clinical outcome [3, 23]. Thus, some authors hypothesized that poor rotational control may predispose patients to future graft failure and need for revision surgery.

Unfortunately, there is a lack of evidence-based treatment algorithms and consensus within the orthopedic community regarding the rotational laxity in primary and recurrent ACL ruptures. One of the underlying problems is that the objective and standardized determination of the internal rotational instability is still difficult. The clinical evaluation encompasses the dial and pivot-shift test. These tests are rater-dependent and influenced by knee position as well as examiner-induced motion. Consequently, the interrater reliability of the manual examination methods is limited [29, 39, 40]. Some of the instrumented measurements require an elaborate set-up including fluoroscopic [28, 30] or magnetic resonance imaging [11, 12]. Other torsion measurements apply the torque to the foot without locking the ankle, which can lead to a considerable overestimation of the rotational laxity [2, 44].

The aim of the current study was to objectify the rotational knee laxity with a validated measurement method, the combined measurement of internal rotation and antero-posterior (a.p.) translation [26], and to compare primary ACL ruptures with recurrent instabilities after previous ACL reconstruction. It is hypothesized that knees with a recurrent instability secondary to ACL reconstruction feature a larger internal rotation laxity than knees with a primary ACL rupture.

## Materials and methods

**Fig. 1:**
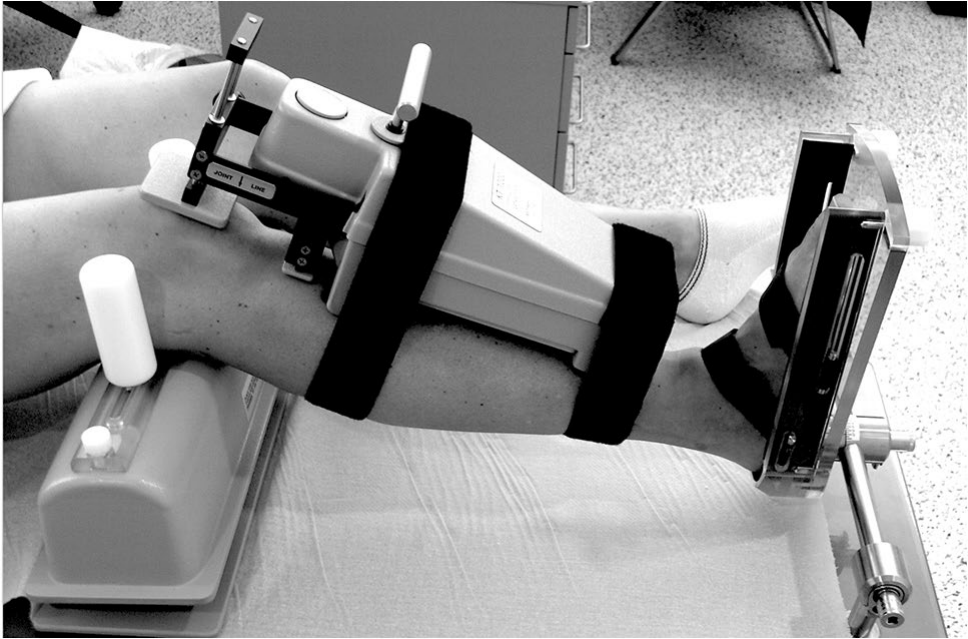
The rotational knee laxity was measured with a validated instrument (Laxitester^®^). The femur was fixed at a knee flexion angle of 30° by medial and lateral counter-bearings at the femoral epicondyles. The foot was fixed in a precisely adjustable footplate. The ankle was locked by dorsiflexion using the trapezoidal shape of the talus. Rotation of the lower leg was performed by torque on the footplate. Under these conditions, the torque applied to the foot is transmitted to the lower leg (25)

This clinical comparative study was conducted investigating patients with primary ACL rupture and patients with ACL rerupture secondary to ACL reconstruction. Knee joints were evaluated clinically and by instrumented measurement of the internal rotational laxity and antero-posterior laxity with a validated Instrument Laxitester^®^ (ORTEMA Sport Protection, Markgroeningen, Germany) and KT1000^®^ (MEDmetric Corporation, San Diego, CA) [26], respectively (Fig. 1). Included were voluntary consecutive patients of the clinic with primary unilateral ACL rupture and patients with ACL rerupture, 20–55 years of age, ASA classification I or II and a BMI of 18–30 after differentiated information and counseling. Exclusion criteria were collateral ligament instabilities > I° (2–5 mm), additional posterior instabilities, axis deviation (varus or valgus) of more than 5°, and knee osteoarthritis grade 2 or higher according to the Kellgren and Lawrence classification [21].

The clinical data were assessed utilizing the IKDC 2000 subjective form, the Lysholm Score, and the Tegner Activity Scale [17, 20]. Conventional X-rays in two planes, a tangential view of the patella in 45° knee flexion and a long leg standing radiograph were made in terms of the group’s clinical routine and in addition to the clinical examination. The clinical examination encompassed a thorough evaluation of the collateral and the posterior cruciate ligaments in order to screen for exclusion criteria. With regard to the aim of the study, the pivot shift phenomena were determined subdividing the grades of laxity glide ( +), clunk (+ +), and gross (+ + +). The clinical examination was carried out by two independent orthopedic and trauma surgeons with specific experience of more than ten years. The investigators were blinded. The study was conducted as a single centre study and the patients were examined in the outward patient clinic of the group’s hospital.

The rotational knee laxity was measured with a validated Instrument (Laxitester^®^, Fig. 1). The femur was fixed at a knee flexion angle of 30° by medial and lateral counter-bearings at the femoral epicondyles. The foot was fixed in a precisely adjustable footplate. The ankle was locked by dorsiflexion using the trapezoidal shape of the talus. Rotation of the lower leg was performed by torque on the footplate. Under these conditions, the torque applied to the foot is transmitted to the lower leg. Internal and external rotation angles of the lower leg were determined with a torque of 2 Nm. The previous validation study showed that a torque of more than two Nm is perceived as painful by the patient even if the knee is not irritated and leads to increased muscular tension. The accuracy of the device has been described to be 5° [26]. In addition, a.p. translation was measured using the KT1000^®^ Arthrometer in neutral position of the lower leg as well as in internal and external rotation as previously described [24, 25]. The a.p. translation was conducted with the leg being fixed in the Laxitester^®^. In order to allow for comparison with other instrumented studies and to exclude pretensioning of the gastrocnemius muscle, the a.p. translation was measured in a neutral ankle position. All other measurements were performed in a defined dorsiflexion of the ankle [26]. The instrumented measurements were conducted by a single investigator (G.H.). Each rotation measurement was carried out and recorded 3 times. All values are listed in Table 4. The mean of the side-to-side difference and the standard deviation were included in the further calculation. The side-to-side difference of the internal rotation angle of the knee joint under 2 Nm torque was set as the primary endpoint. Secondary endpoints were the grading of the pivot-shift phenomenon, the a.p. translation measured by the KT1000^®^ Arthrometer, the IKDC 2000 subjective form, the Lysholm Score, and the Tegner Activity Scale.

### Statistical evaluation

Means and standard deviations were calculated for all numeric values. The Kolmogorov–Smirnov test was applied to check for normal distribution. In order to test for significance, the Student's *t* test was applied for numeric variables. The chi-square test was used to test for significant differences in distribution of categorical variables. The Spearman’s correlation coefficient (r_s_) was calculated to measure the strength of a linear correlation between two variables. Cohen's kappa coefficient (*Κ*) war used to measure inter-rater reliability for categorical items. The Pearson’s correlation coefficient (*r*) was applied to measure of the degree of linear relationship between sets of numeric variables.

Power calculation: With a significance level of 5% for two-sided testing, a power of 80% and an expected difference of 10° with a standard deviation of 10°, 20 test subjects per group are required to obtain a statistically significant difference in the internal rotation angle of the knee joint in the side-to-side comparison. Differences with a *p* value < 0.05 were regarded as statistically significant. Data analysis was performed by SPSS^®^ statistical software version Premium 26 (SPSS, Chicago, USA).

### Institutional review board approval

Freiburg University authorities gave the application number EK-Freiburg 542/19 the institutional review board (IRB) approval on February 25, 2020.

## Results

### Study cohorts

Twenty-four patients with primary ACL rupture and 23 patients with rerupture after ACL reconstruction were included in this clinical comparative study. None of the patients that qualified for inclusion declined consent and participation. The patients’ demographics and injury data are presented in Table 1. The distribution of meniscus and cartilage damage in both groups is shown in Tables 2 and 3. In the rerupture group a previous suture or a partial resection of the medial meniscus was evident in two and eight cases, respectively. With regards to the lateral meniscus, a previous suture or a partial resection was evident in three and two cases, respectively. Patients with primary ACL rupture showed no significant age difference compared to patients with ACL rerupture (31.3 ± 9.1 and 31.2 ± 6.7 years, *p* = 0.98). No significant difference in gender distribution was detected (*p* = 0.9). Both groups encompassed a limited number of patients with delayed presentation after the trauma (primary ACL rupture = 5; ACL rerupture = 3). With regards to those patients presenting within 6 months after the trauma, no significant differences were found in the interval between trauma and clinical evaluation across the two groups (58.8 ± 34.0 and 50.8 ± 47.5 days; *p* = 0.58). Each rotation measurement was carried out and recorded 3 times. All values are listed in Table 4.

**Table 1:** Patients’ demographics and injury data

	Primary ACL rupture (*n* = 24)	ACL rerupture (*n* = 23)
Age (years)	31.3 (± 9.1)	31.2 (± 6.7)
Gender (m: f)	14: 10	13: 10
Injury mechanism	Soccer = 8 Ski alpine = 5 Handball = 3 Accident = 3 Fall = 2 Others = 3	Adequate trauma (sport injury, accident, fall, etc.) = 10 Inadequate trauma (an incident during minor movement or everyday stress) = 5 Chronic instability (no history of a specific incident) = 8
Clinical evaluation a) ≤ 180 days after trauma^a^ b) > 180 days after trauma c)w/o history of trauma	*n* = 19 *n* = 5 *n* = 0	*n* = 12 *n* = 3 *n* = 8
Interval trauma—clinical evaluation in group a) (days)	58.8 (± 34.0)	50.8 (± 47.5)

*m* male, *f* female

^a^Encompasses adequate and inadequate traumata in the group of ACL rerupture

**Table 2:** The distribution of meniscus damage in both groups

Meniscal lesion	Primary ACL rupture (*n* = 24)		Rerupture after ACLR (*n* = 23)	
	Medial	Lateral	Medial	Lateral
None	18	13	12	16
Moderate	3	5	4	2
Severe	3	6	7	5

**Table 3:** The distribution of cartilage damage in both groups

Chondro-malacia	Primary ACL rupture (*n* = 24)			Rerupture after ACLR (*n* = 23)		
	Medial	Lateral	PF	Medial	Lateral	PF
0	17	20	18	10	16	15
I/II	3	2	5	7	5	6
III	4	2	1	4	2	2
IV	0	0	0	2	0	0

**Table 4:** Each rotation measurement was carried out and recorded 3 times

A	Primary ACL rupture				ACL rerupture			
	1st run	2nd run	3rd run	Mean	1st run	2nd run	3rd run	Mean
∆ IRO	0	− 5	5	0	10	5	15	10
	0	0	− 5	− 1.7	15	15	20	16.7
	0	0	5	1.7	0	− 5	5	0
	0	5	− 5	0	5	0	10	5
	0	5	0	1.7	10	15	0	8.3
	5	10	5	6.7	5	10	0	5
	0	5	5	3.3	15	10	20	15
	0	0	0	0	25	30	20	25
	5	10	0	5	0	0	5	1.7
	0	5	0	1.7	0	− 5	5	0
	0	0	5	1.7	10	15	5	10
	10	10	15	11.7	0	5	0	1.7
	0	0	5	1.7	10	10	15	11.7
	10	10	5	8.3	10	15	10	11.7
	0	0	0	0	0	0	0	0
	10	15	5	10	0	5	− 5	0
	0	0	0	0	10	10	5	8.3
	15	15	20	16.7	35	30	40	35
	5	5	0	3.3	10	5	5	6.7
	0	0	5	1.7	0	− 5	0	− 1.7
	5	0	5	3.3	15	10	10	11.7
	0	0	0	0	5	10	10	8.3
	0	0	5	1.7	10	10	10	10
	10	5	10	8.3				
Mean (± SD)	3.1 (± 4.6)	4.0 (± 5.3)	3.8 (± 5.6)	3.6 (± 4.5)	8.7 (± 8.7)	8.5 (± 9.3)	8.9 (± 9.8)	8.7 (± 8.6)

The mean of the side-to-side difference and the standard deviation were included in the further calculation

A) *IRO* internal rotation

B) *ERO* external rotation

### Instrumented measurement of anterior and internal rotational laxity

**Fig. 2:**
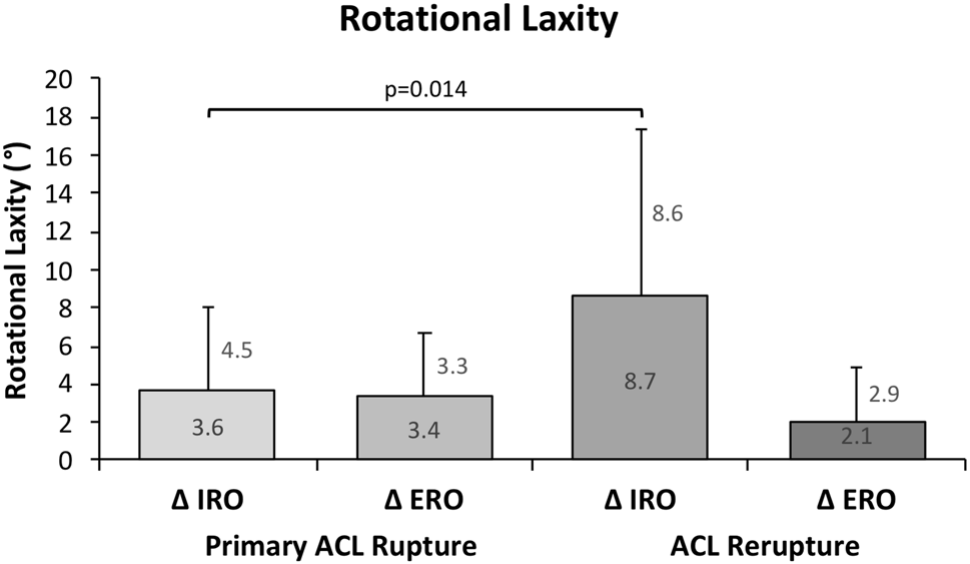
A significant side-to-side difference of internal rotation laxity was found with a mean of 8.7 ± 8.6° in patients with an ACL rerupture as compared to a mean of 3.6 ± 4.5° in patients with a primary ACL rupture (*p* = 0.014). *IRO* internal rotation, *ERO* external rotation

The side-to-side difference in anterior translation was measured by means of the KT1000^®^ and revealed 4.0 ± 1.7 mm in patients with primary ACL rupture as well as 4.0 ± 1.8 mm in patients with ACL rerupture. This difference was statistically not significant (*p* = 1.0). In contrast, a significant side-to-side difference of internal rotation angles was found with a mean of 8.7 ± 8.6° in patients with ACL rerupture as compared to a mean of 3.6 ± 4.5° in patients with primary ACL rupture (*p* = 0.014; Fig. 2). A significant side-to-side difference of external rotation angles was not seen across the two groups (*p* = 0.18; Fig. 2). The individual rotation measurements are provided in Table 2. The relationship between the rotation measurements showed a strong positive correlation with a Pearson correlation coefficient of *r* = *0.847.* A side-to-side difference of internal rotational laxity ≥ 10° was found significantly more frequent in patients with ACL rerupture as compared to patients with primary ACL rupture with 53.6% versus 19.4% of the measurements, respectively (*p* < 0.001).

### Pivot-shift phenomenon

All patients of both groups revealed a positive pivot-shift test for the injured knees. The distribution of the grades of the pivot-shift phenomenon showed significant differences between patients with primary ACL tears and ACL rerupture (*p* < 0.001).

**Fig. 3:**
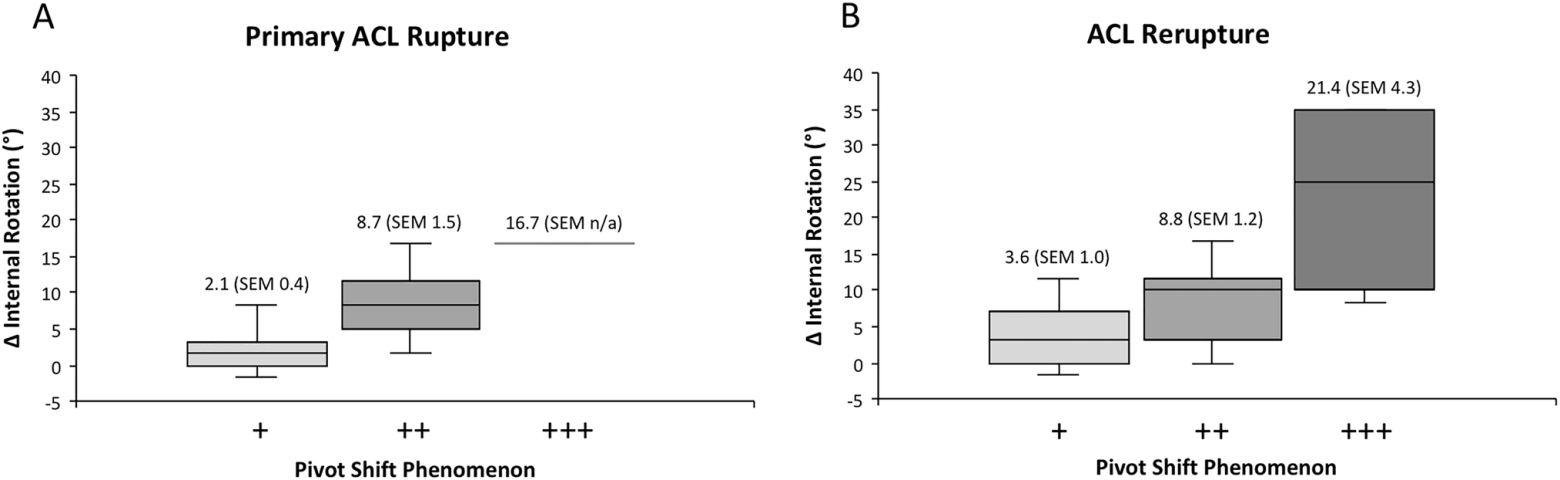
A highly significant relationship between the extent of the pivot-shift phenomenon and side-to-side difference of internal rotation could be demonstrated (primary ACL tear r_s_ = 0.695; *p* (*R*^2^) < 0.001; ACL rerupture: r_s_ = 0.637; *p* (*R*^2^) < 0.001)

**Fig. 4:**
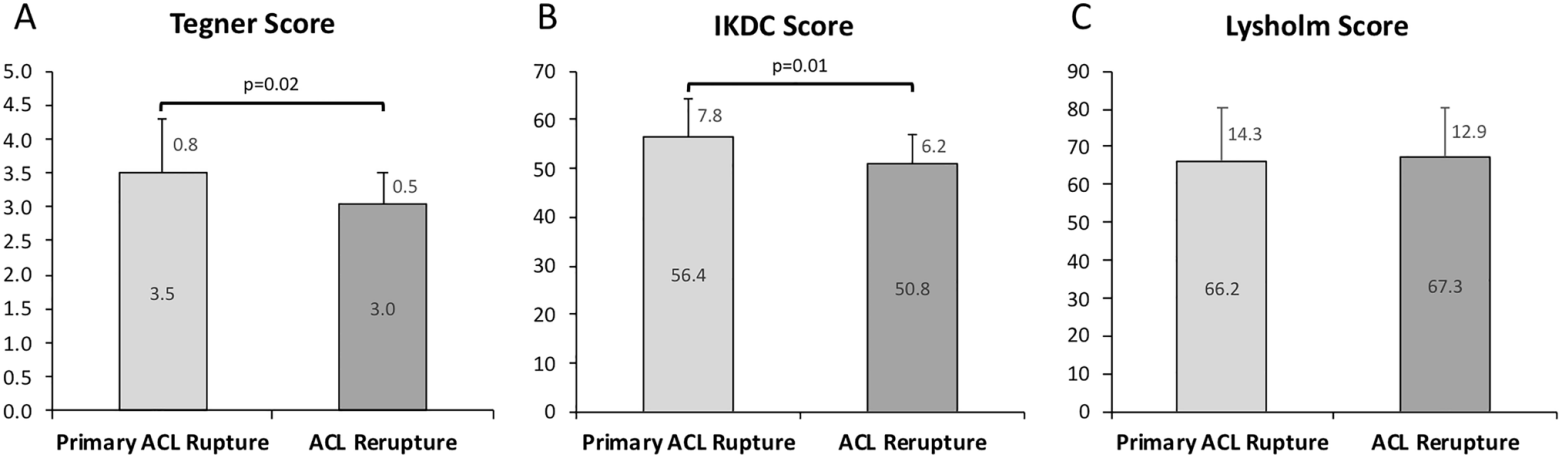
Patients with a primary ACL tears scored significantly higher in the Tegner Activity Scale (*p* = 0.02) and the IKDC 2000 subjective questionnaire (*p* = 0.01) as compared to patients with an ACL rerupture. There was no significant difference in the Lysholm Score (*p* = 0.78)

In patients with primary ACL tears pivot-shift glide (+) was found in 38 tests, pivot-shift clunk (+ +) in 9 tests, and pivot-shift gross (+ + +) in 1 test as compared to 18, 21, and 7 tests in the group of ACL reruptures, respectively. Cohen's weighted *Κ* (quadratically) calculated for the pivot-shift result of two raters on 47 cases resulted in a *Κ* = 0.445 as sign for a moderate agreement. A highly significant relationship between the extent of the pivot-shift phenomenon and side-to-side difference of internal rotation could be demonstrated (primary ACL tear r_s_ = 0.695; *p* (*R*^2^) < 0.001; ACL rerupture: r_s_ = 0.637; *p* (*R*^2^) < 0.001) (Fig. 3). No correlation was found between the amount of side-to-side difference of the anterior translation measured by KT1000® and the extend of side-to-side difference in internal rotational instability in patients with primary ACL ruptures (R = − 0.284; p = 0.178) and patients with ACL rerupture (*R* = 0.367; *p* = 0.086). Patients with a primary ACL tears scored significantly higher in the Tegner Activity Scale (*p* = 0.02) and the IKDC 2000 subjective questionnaire (*p* = 0.01) as compared to patients with an ACL rerupture. There was no significant difference in the Lysholm Score (p = 0.78) (Fig. 4).

(*R* = − 0.284; *p* = 0.178) (*R* = 0.367; *p* = 0.086).

### Patients reported outcome measures (PROM)

Patients with primary ACL tears scored significantly higher values in the Tegner Activity Scale with a mean of 3.5 ± 0.8 points compared to patients with ACL rerupture with a mean of 3.0 ± 0.5 points (*p* = 0.02). The IKDC 2000 subjective questionnaire revealed significantly better scores in patients with primary ACL tear compared to patients with ACL rerupture (56.4 ± 7.8 vs. 50.8 ± 6.2; *p* = 0.01). No statistically significant differences were observed between patients with primary ACL tear and ACL rerupture when comparing the results of the Lysholm Score (66.2 ± 14.3 vs. 67.3 ± 12.9; *p* = 0.78).

## Discussion

This is the first study revealing a significantly higher internal rotation laxity in knee joints with recurrent ACL rupture secondary to reconstruction compared to primary ruptures of the native ACL. The study further demonstrates that the Laxitester^®^ (Fig. 1) is a feasible measuring device that allows for precise quantification of rotational laxity and reliable identification of patients with increased or excessive internal rotation instability. The repeated measurements show a strong positive correlation with a Pearson’s correlation coefficient of *r* = 0.847. In future, these instrumented measurements can help in decision making on which ACL reconstruction and revision may benefit from additional external anterolateral tenodesis.

The Laxitester^®^ identifies increased internal rotation laxity in both, primary and recurrent ACL ruptures. However, across the two cohorts, the mean side-to-side difference of internal rotation laxity is significantly higher in the rerupture group (8.7 ± 8.6° vs. 3.6 ± 4.5°, *p* = 0.014; Fig. 2). With regards to the primary ACL rupture, the present findings go well in line with Moewis et al., who reported a mean side-to-side difference of 5° internal rotation laxity when comparing primary ACL ruptured and healthy knees exposed to a torque of 2.5Nm in 30° flexion [28]. On the other hand, the physiological maximum side-to-side difference in healthy knees does not seem to exceed 1.5° [1]. However, no study has yet systematically investigated the extend of internal rotational laxity after ACL rerupture secondary to ACL reconstruction.

For the purpose of this study, a side-to-side difference of ≥ 10° of internal rotation laxity was thought to be clinically relevant. This cutoff was set in agreement with the previous publications on the dial test [40] and knee biomechanics [42, 44, 47] that also defined a side-to-side difference of ≥ 10° as a pathologic internal rotation laxity. A side-to-side difference of internal rotational instability ≥ 10° was found significantly more frequent in patients with ACL rerupture compared to patients with primary ACL rupture with 19.4% versus 53.6% of the measurements, respectively (*p* < 0.001). The increased internal rotation laxity is most likely a result of an additional injury to the anterolateral capsule and the ALL [15, 16, 32, 43]. Unfortunately, the incidence of additional relevant injuries to the ALL, in primary as well as in recurrent ACL ruptures, is still largely unknown. In contrast to the present findings revealed by instrumented laxity measurements, the magnetic resonance imaging (MRI) study by Carr et al. [5] did not find significant differences in the frequency of partly or completely torn ALL when comparing primary and recurrent ACL ruptures. Nevertheless, in both cases the incidence of lesions to the ALL was over 50%. In general, such MRI findings do not reliably allow for conclusions on the actual clinical laxity.

The clinical evaluation of the extend of the pivot-shift phenomenon depends on the examiner and features limitations in the reliability. Noyes et al. [31] found that the magnitude of anterior subluxation varied greatly between examiners due to differences in the technical implementation. A meta-analysis of 28 studies showed that the pivot-shift test has a high specificity of 98%, but only a sensitivity of 24% [4]. In the present study the pivot-shift test carried by two blinded raters revealed a Cohen's weighted *Κ* of 0.445 as sign for a moderate agreement.

Nevertheless, the present study revealed a strong correlation of the pivot-shift phenomenon and the instrumented laxity measurements (primary tear r_s_ = 0.695; rerupture r_s_ = 0.637; *p* (*R*^2^) < 0.001 in both groups). In patients with increased risk of failure, some groups base their indication for simultaneous ALL augmentation during primary ACL reconstruction on the grade of pivot-shift phenomenon [14, 16, 37, 43]. Taking a closer look at the present findings suggests a weaker correlation for the excessive internal rotation laxity. While laxities beyond a gross pivot-shift phenomenon cannot be identified clinically, the Laxitester^®^ further distinguishes excessive rotational laxity by providing numeric values. The sound inter- and intrarater agreement [26] and the precise numeric determination of the rotational laxity are clear strongpoints of the instrumented measurements. The Laxitester^®^ reliably identifies patients with increased and excessive internal rotation laxity. These patients may benefit in particular from simultaneous ALL augmentation during primary or revision ACL reconstruction to further improve the rotational stability [45]. On the other hand, unnecessary simultaneous ALL augmentation in moderate internal rotational laxity can be avoided, thus, potentially reducing the phenomena of over-constrained stability observed in undifferentiated populations [6].

On average, the patients with a rerupture after ACL reconstruction score significantly lower on the Tegner Activity Scale and the IKDC 2000 subjective form when compared with primary ACL ruptures. The IKDC 2000 subjective questionnaire revealed a mean of 56.4 ± 7.8 in primary ACL tears compared to 50.8 ± 6.2 in ACL reruptures (*p* = 0.01). A recent systematic review and meta-analysis reported comparably low mean IKDC sores of 43.9 to 51.4 in patients with recurrent ACL rupture [9]. The differences in the Lysholm score did not reveal to be statistically significant between the two groups. While primary ACL tears showed a mean of 66.2 ± 14.3, ACL reruptures averaged in 67.3 ± 12.9 (*p* = 0.78). This is comparable to Lysholm scores reported by Weiler et al., who revealed a mean value of 65 ± 17 in a cohort of 50 ACL reruptures [46]. In addition to the consensus, that lower PROM scores in patients with an ACL rerupture are due to higher numbers of previous operations as well as more pronounced wear and tear of menisci and joint surface cartilage [27], the present study indicates that lower PROM scores may also, at least in part, be due to a higher extend of internal rotation laxity of the injured knee joint.

### Limitations

Some general limitations of the present study result from the design of a clinical comparative study. A specific limitation is the KT-1000^®^, which had widely been utilized by knee surgeons and physiotherapists in the last decade [18], but which to today´s standards is an outdated measuring device with limited accuracy. Another limitation is the accuracy of 5° of the Laxitester^®^.

### Conclusion

Patients with ACL rerupture feature significantly higher internal rotation laxity of the knee compared to primary ACL rupture. The extend of internal rotation laxity can precisely be quantified by means of instrumented measurement. The quantification of internal rotational laxity can facilitate the decision-making process on simultaneous anterolateral ligament augmentation during revision ACL surgery.
